# A Scalable Bidimensional Randomization Scheme for TLC 3D NAND Flash Memories

**DOI:** 10.3390/mi12070759

**Published:** 2021-06-27

**Authors:** Michele Favalli, Cristian Zambelli, Alessia Marelli, Rino Micheloni, Piero Olivo

**Affiliations:** 1 Dipartimento di Ingegneria, Università degli Studi di Ferrara, Via G. Saragat 1, 44122 Ferrara, Italy; michele.favalli@unife.it (M.F.); piero.olivo@unife.it (P.O.); 2 Freelance Consultant, Via Don Pino Puglisi 4, 24048 Treviolo, Italy; alessiamarelli@gmail.com; 3 Freelance Consultant, Via Roma 23, 22010 Moltrasio, Italy; rino.micheloni@ieee.org

**Keywords:** 3D NAND Flash, RBER, reliability, flash signal processing, randomization scheme

## Abstract

Data randomization has been a widely adopted Flash Signal Processing technique for reducing or suppressing errors since the inception of mass storage platforms based on planar NAND Flash technology. However, the paradigm change represented by the 3D memory integration concept has complicated the randomization task due to the increased dimensions of the memory array, especially along the bitlines. In this work, we propose an easy to implement, cost effective, and fully scalable with memory dimensions, randomization scheme that guarantees optimal randomization along the wordline and the bitline dimensions. At the same time, we guarantee an upper bound on the maximum length of consecutive ones and zeros along the bitline to improve the memory reliability. Our method has been validated on commercial off-the-shelf TLC 3D NAND Flash memory with respect to the Raw Bit Error Rate metric extracted in different memory working conditions.

## 1. Introduction

The 3D NAND Flash technology is the primary choice for non-volatile mass storage platforms such as Multimedia Cards (MMCs) and Solid State Drives (SSDs) [1]. Compared with its planar predecessor, this technology offers a significantly higher storage density and better scaling features [2,3]. From the reliability standpoint, the 3D NAND Flash technology inherits the issues already documented for planar NAND Flash, such as wear-out failures due to repeated data writing/erasing (i.e., endurance failures [4]), high temperature sensitivity either in static (i.e., data retention [5]) or in dynamic (i.e., cross-temperature [6]) working conditions, and disturbances due to frequent access to the memory (e.g., read disturb [7]). On top of these, novel reliability threats specifically belonging to the physical nature of 3D devices come into play [8,9]. At the system level, all these reliability detractors are perceived through an increase of the Fail Bits Count (FBC) exposed by the 3D NAND Flash after operation. An efficient way to handle the ever-growing FBC during the entire memory lifetime is to rely on complex Error Correction Code (ECC) engines [10] that work on a translation of the FBC concept into an equivalent Raw Bit Error Rate (RBER) to perform the error recovery. However, the RBER is highly dependent on the pattern applied to write the data on the memory; therefore, without decoupling it from the intrinsic 3D NAND Flash reliability, we would experience some unfortunate situations where RBER is higher than the Shannon’s limit [11].

A fundamental component in storage systems, the goal of which is to avoid these events, is the randomizer. This object ensures that the memory data programming is achieved in the most efficient way, making the probability of a worst-case data pattern statistically negligible. The idea behind data randomization is to perform a transformation from original user data by simply inserting an exclusive OR (XOR) operation between the data path and the output of a Linear Feedback Shift Register (LFSR) initialized by a seed [12,13]. The seed is the starting value to be loaded into the LFSR to enable the generation of random patterns. The value of the seed is usually selected to avoid pattern repetitions and must be greater than zero to start the generation of pseudo-random sequences. Additionally, multiple seeds can be exploited to reduce the correlation effects between different LFSRs’ random number generations. The randomizer block can be implemented either on-chip in the circuit periphery close to the memory array [14,15], or off-chip by implementing its function in the storage controller when its architectural complexity requires additional logical operations, such as for example, those required for seed generation [16]. Despite the importance of this component, we must note that the focus of these implementations is on the random value patterns’ generation primarily in the horizontal dimension (i.e., page- or wordline-wise), while being less effective in the vertical dimension (i.e., string- or bitline-wise) of the memory. Most randomization schemes do not bother with the maximum number of consecutive ones or zeros along the bitline that could impair the sensing operation, thus resulting in a localized RBER increase.

In the literature, cumbersome methodologies based on multiple chained LFSRs, or even on look-up tables exploited for seed generation with arithmetic functions based on heuristics, are adopted [16]. However, all the proposed solutions lack information in terms of the mathematical approach required to achieve randomization. From the storage system designer point of view, this will be a limiting factor since every time there is a technology update of the storage medium (e.g., a change to the memory density, storage paradigm, etc.), there will be a forced change of the randomization scheme.

In this work, we tackle the data randomization challenge in Triple Level Cell (TLC) 3D NAND Flash memories by presenting a simple yet scalable bi-dimensional randomizer that guarantees both the horizontal and the vertical randomization while defining an upper bound on the maximum sequence length of consecutive ones and zeros along the dimensions.

The contributions of this paper can be summarized as follows:

1. We show that a chained structure of two *k*-bits LFSRs can provide, from a statistical standpoint, both the horizontal and vertical data randomization while guaranteeing a *k*-bit upper bound on the maximum sequence length of consecutive ones and zeros;
2. We show that our proposed randomization scheme introduces a low-complexity hardware overhead, most of which scales automatically with the memory size and is independent of cumbersome heuristics, to achieve seed randomization or look-up tables (LUTs) for seed storage, to potentially be adopted by different memory technologies and vendors;
3. We benchmark the effectiveness of our scheme by measuring the RBER characteristics of a Triple Level Cell (TLC) 3D NAND Flash memory during both endurance and data retention stress.

## 2. Background

### 2.1. 3D NAND Flash Memory Architecture and Randomization Fundamentals

**Figure 1:**
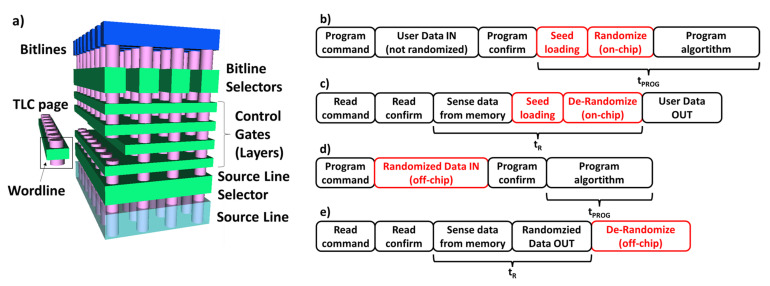
(**a**) The TLC 3D NAND Flash architecture. Reprinted with permission from [9] under Creative Commons License 4.0 (CC-BY). (**b**) Sequence of operations during program operation considering on-chip randomization. (**c**) Sequence of operations during read operation considering on-chip randomization. (**d**) Sequence of operations during program operation considering off-chip randomization. (**e**) Sequence of operations during read operation considering off-chip randomization.

The architecture of a 3D NAND Flash is described in the sketch in Figure 1a. The primary element of the array is the stack of Control Gates (CGs), also indicated as Layers. Associated with each CG, there are several wordlines that depend on the specific integration concept for the memory [17]. The bottom of the memory architecture is represented by the Source Line and the Source Line Selectors of the 3D NAND Flash string. Multiple holes are drilled through the CG stacks and plugged with poly-silicon in order to form a series of vertically arranged 3D NAND Flash memory cells. In TLC architectures, all the cells belonging to a wordline can store up to three bits per cell, defined as Lower Significant Bit (LSB), Central Significant Bit (CSB) and Most Significant Bit (MSB); Bitline selectors and bitline (BL) contacts are on top of the structure.

The goal of the data randomization is that this operation will scramble the data to be sent for programming in the different memory wordlines after the data input from the host interfaces with the memory, and the de-randomization operation happens before the data output from the memory to the host starts [12,14]. Figure 1b,c shows the operation flow, considering the case of an on-chip implemented randomizer. The random seed is loaded into an internal circuit of the 3D NAND Flash memory, called a *page buffer*, via the memory data-path. Then, additional circuits take the seed and execute bit-wise XOR of the original data input from the host and random sequence in the page buffers. The program algorithm can then start. On the contrary, de-randomizing operations happen during read mode: first, the data from the memory are sensed, then the seed is loaded into a page buffer and, finally, a bit-wise XOR of sensed data and random sequence is executed making the original data available to the host. In the case of on-chip randomizers, the time for on-chip randomizing is added to the program ($t_{PROG}$)/read ($t_{R}$) time. Off-chip randomizers can help to reduce the former times by providing a scrambled version of the data to be programmed in the memory, but in this case, it is the host that needs to take care of both randomization and de-randomization (see Figure 1d,e).

### 2.2. Randomizers Based on LFSRs

**Figure 2:**
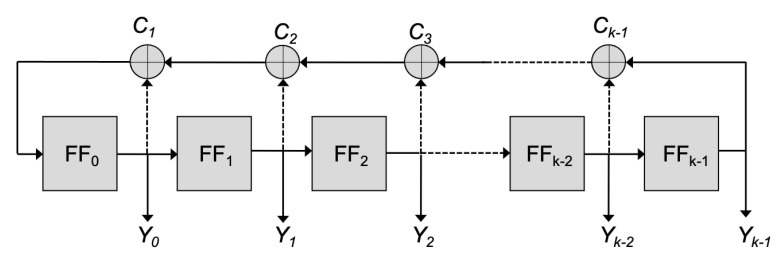
Schematic representation of an ALFSR. It is realized by *k* D-flip-flop (FF${}_{0}÷$ FF${}_{k-1}$) and a feedback path where some XOR $C_{i}$ may be present. The feedback function depends on the presence/absence of the XOR (at least one must be present). Preset signals for register initialization are not shown. The autonomous property indicates that no input is present, so that once initialized in any state but all zeros, the cycling diagrams depend only on the feedback function.

The principal solutions adopted for data randomization utilize a *k*-bit ALFSR (Autonomous Linear Feedback Shift Register), as shown in Figure 2. Feedback functions exist for any *k* value [18], guaranteeing that, once initialized in any state but “all zeros”, the register evolves through all the $2^{k}-1$ states before returning to the initial state.

The initial state is generally denoted as the register *Seed*. If the sequence generated by the ALFSR—for instance, that collected at exit $Y_{k-1}$—is sufficiently long, pseudorandom characteristics are guaranteed: the probability of having bits equal to 0 is 0.5, that of having any 2-bit sequence (00, 01, 10, 11) is 0.25, that of having any 3-bit sequence (000, 001, ⋯, 111) is 0.125 and so on.

Besides these important statistical properties, for the problem at hand it must be observed that the maximum number of consecutive zeros is equal to k−1, whereas the maximum number of ones is equal to *k*. The former statement derives from the fact that *k* consecutive zeros correspond to the “all zeros” register state, which does not belong to the state diagram (otherwise the register would remain in that state because of the absence of any input). The latter derives from the fact that, when *k* consecutive bits equal to one are encountered, the ALFSR is in the “all one” state and, consequently, the next state must be 0111 ⋯ 111 (otherwise the ALFSR would remain in the same state, contradicting its cycling properties). The sequences of k−1 zeros and those of *k* ones occur just once in an entire $2^{k}-1$ bit sequence.

**Figure 3:**
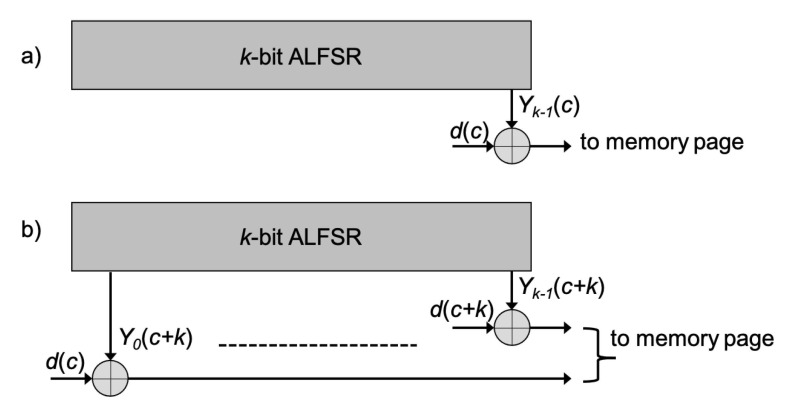
Possible schemes used to randomize data in a memory page. (**a**) at any clock cycle *c*, the input data d(c) is XORed with the last register bit $Y_{k-1}\left(c\right)$; (**b**) *k* clock pulses are applied to the register, then the register content is XORed with *k* input data and the procedure is repeated until all page data are randomized.

Two possible schemes adopted to randomize the data to be stored in a memory page are shown in Figure 3. In the former, at any clock cycle *c*, input data d(c) is XORed with the content of the last register bit $Y_{k-1}\left(c\right)$; in the latter, the ALFSR is cycled for *k* clock cycles, then all the register content $Y_{0}\left(c+k\right)$, ⋯, $Y_{k-1}\left(c+k\right)$ is XORed with *k* input data (d(c), ⋯, d(c+k)) and this procedure is repeated until all page data are randomized. If the same Seed in considered, the two schemes are fully equivalent in terms of randomization since, for the same data input sequence, they produce the same data sequence to be stored in the memory page.

A 3D NAND Flash memory block is constituted by $N_{P}$ pages featuring $N_{B}$ cells each. $N_{B}$ is in the range of 4 kB ÷ 16 kB (corresponding to $2^{15}÷2^{17}$ cells) whereas $N_{P}$ is in the range of 256 ÷ 1024 (i.e., $2^{8}÷2^{10}$). The typical ALFSR length adopted in the randomizing schemes is k=32, so that a sequence of $N_{L}$ = $2^{32}-1$ can be generated by the register before returning to its initial state. Since $N_{B}\ll N_{L}$, it is clear that the statistical properties of the ALFSR are not fully exploited. A 32-bit ALFSR is generally considered a good player in data randomization.

The same ALFSR is used to randomize data for all pages in a block by changing its Seed for each page. The different Seeds can be picked from an LUT and then stored in a table, or generated internally by manipulating the page address, depending on the strategy adopted by the memory manufacturer. Unfortunately, this technique, even if providing a relatively good randomization for data stored in a page, fails at guaranteeing a good vertical data randomization along the bitline.

To illustrate the problem, Table 1 shows the sequences generated by a 4-bit ALFSR considering $N_{B}$ = $N_{P}$ = 15, obtained by randomly picking the initial Seed. As can be seen in the 4th column, all ones and all zeros are clustered, confirming that, whereas ALFSRs can be conveniently used to randomize data in the horizontal direction, no statistical predictions can be drawn when looking at a single bitline.

To analyze the problem in real cases, we performed simulations using a 32-bit ALFSR considering $N_{P}=256$ pages, each of $N_{B}=2^{17}$ cells (i.e., 16 kB). Two procedures have been selected to determine the ALFSR’s Seeds: in the former, each Seed is a linear manipulation (7∗p+1) of the page address *p*; in the latter, each Seed is picked randomly among all the $2^{32}-1$ possibilities.

**Figure 4:**
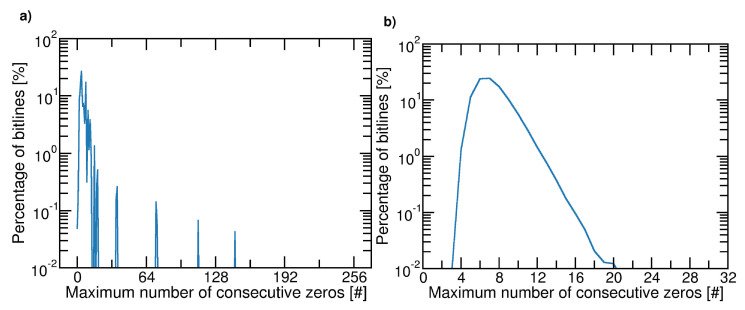
Percentage of bitlines as a function of the maximum number of consecutive zeros in a bitlines. Analysis has been performed considering a 32-bit ALFSR on a memory array of $N_{P}=256$ pages and $N_{B}=2^{17}$ cells. Case (**a**): Seeds derived from a mathematical manipulation of the page address; case (**b**): seeds generated randomly.

**Figure 5:**
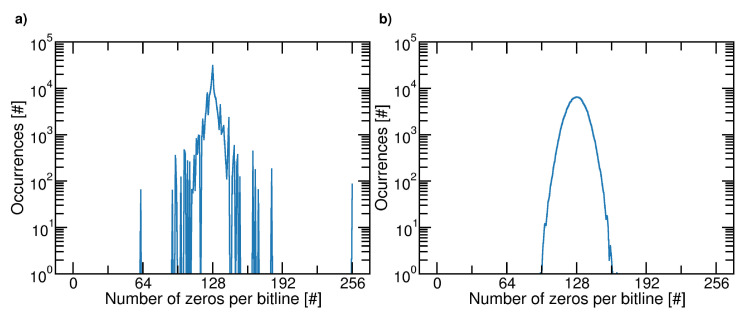
Distributions (occurrences) of the zeros probability in a bitline. Analysis has been performed considering a 32-bit ALFSR on a memory array of $N_{P}=256$ pages and $N_{B}=2^{17}$ cells. Case (**a**): Seeds derived from a mathematical manipulation of the page address; case (**b**): seeds generated randomly.

Figure 4 shows, for the two cases, the frequency of the maximal length of zeros per bitline. Similar results are expected for ones. It must be noted that, in the first case, we observe more than 128 consecutive zeros along the bitline, thus resulting in potential reliability issues for the 3D NAND Flash memory. Figure 5 shows the distributions of the probabilities of zeros in a bitline for the two cases. Once again, we observe that the first case is critical since there are some bitlines where the number of ones and zeros is strongly unbalanced.

When the ALFSR Seeds are mathematically derived from the address page, the probability of producing a specific maximum length cluster shows a discrete spectrum: in particular, in 85 bitlines, all data are zeros. On the contrary, with the set of randomly selected Seeds used in this case, no clusters longer than the ALFSR length are found. However, it can be verified that, since the probability of zeros in a bitline shows a Gaussian-like distribution, the length of zero runs, and the probability of zeros per bitline may range over all their possible values.

However, since generally $N_{P}\ll 2^{k}-1$, it is possible to simulate the behavior of an ALFSR considering a random Seed for each page and repeat the simulation until a set of $N_{P}$ Seeds is found to guarantee, for each bitline, a number of zeros close to 50% and a predefined maximum length of clusters of consecutive ones or zeros. Then, these Seeds can be stored in an LUT integrated either on-chip or off-chip in the storage controller. Unfortunately, the quality of the set of Seeds depends on $N_{P}$ and on $N_{B}$ so that the procedure determining a good set of Seeds must be repeated from scratch when considering a different memory architecture.

**Figure 6:**
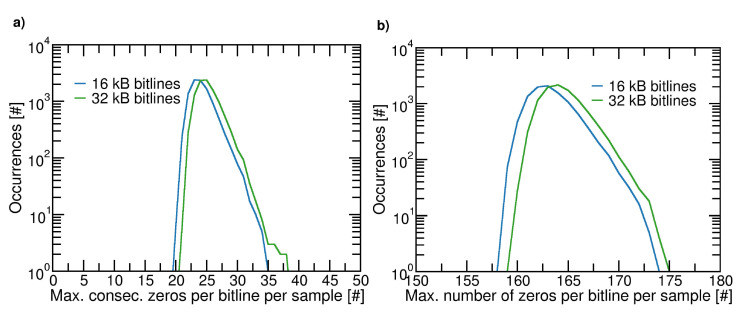
(**a**) Maximum consecutive number of zeros in a bitline per generated sample as a function of the bitlines’ dimension; (**b**) Maximum number of zeros in a bitline per generated sample as a function of the bitlines’ dimension. In these simulations we consider 256 wordlines.

To explore the impact of random seeds selection, we randomly generated 10,000 sets, each of them containing 256 random seeds. Each set is used to feed the initial states of a 32-bit ALFSR that is used to write an array of $N_{P}\times B=256\times 2^{17}$ cells (i.e., 16 kB). For each of these 10,000 arrays, we extracted some worst case statistical parameters, namely: (i) the maximal length of consecutive zeros (ones); (ii) the maximal number of zeros (ones) in a bitline. In case (i), Figure 6a shows the number of arrays featuring a given maximal length. As can be seen, while the mean value is in the interval number of 23–24, outliers are present, featuring runs of more than 30 consecutive values. Figure 6b, instead, shows the number of arrays featuring a given maximum value of zeros. In this case, the figure also shows that outliers exist that feature a number of zeros per bitline that is larger than the average. Moreover, we must note that, as the dimension of the bitlines scales up (32 kB as shown in Figure 6), the formerly defined statistical parameters worsen.

This means that any selected set of random weights should be simulated and possibly discarded to optimize these parameters. Random selected seeds, therefore, do not represent an effective solution for the problem considered in this work. In the remainder of this work, we will propose a randomization scheme independent of the dimension of the bitlines.

## 3. The Proposed Solution

The solution proposed here guarantees the correct data randomization in a memory page and, at the same time, provides an upper bound for the maximum length of clusters of ones and zeros and an almost equal percentage of ones and zeros for all bitlines. The solution can be conveniently described by means of the following example: consider, for the sake of simplicity, a 4-bit ALFSR initialized with a random seed and consider the $2^{4}-1$ bit-long sequence generated as reported in the first row of Table 2. Then, consider the 2nd row as the 1st one left-shifted by one position, the 3rd row as the 2nd one left-shifted by one position and so on. We can observe that the resulting matrix is symmetrical.

By construction, it can be observed that any *i*th column is equal to the *i*th row (for instance, the 5th row and column in Table 2 are highlighted.) Therefore the statistical properties guaranteed in a $2^{k}-1$ long sequence produced by a *k*-bit ALFSR can be found in any column: (i) the number of ones is $2^{k-1}$ whereas that of the zeros is $2^{k-1}-1$; (ii) the length of the maximum sequence of ones is equal to *k* whereas that of the zeros is equal to k−1. In addition, as already stated, the sequence of k−1 zeros and that of *k* ones occurs just once in an entire $2^{k}-1$ bit-long sequence.

### Hardware Realization

**Figure 7:**
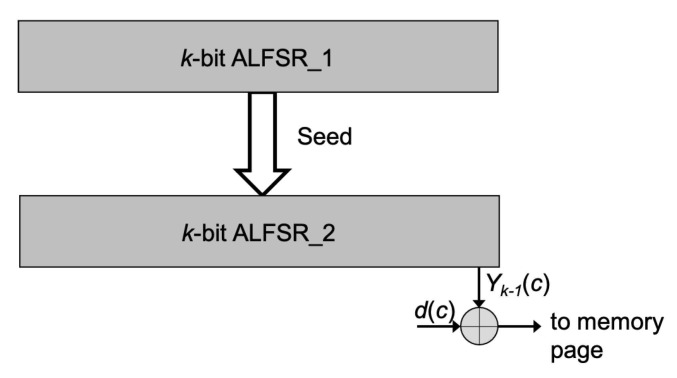
Proposed scheme for data randomization. For each memory page, ALFSR_1 generates the seed initializing ALFSR_2 whose content is used to randomize the data to be stored. $k=\lceil \log_{2}N_{P}\rceil$.

The proposed solution can be easily implemented for any 3D NAND Flash memory architecture. It makes use of two *k*-bit ALFSRs, where $k=\lceil \log_{2}N_{P}\rceil$ (i.e., *k* = 8 for $N_{P}$ = 256; *k* = 9 for 257 < $N_{P}$ ≤ 512, and so on). As shown in Figure 7, one ALFSR is used to generate the Seeds for the second ALFSR, which is used to generate the sequence randomizing the data in a page.

The algorithm to be applied is the following:

1. After a block erase operation, initialize ALFSR${}_{1}$ with *SEED${}_{IN}$*. *SEED${}_{IN}$* can always be the same or, more conveniently to avoid a reliability degradation of 3D NAND Flash cells during endurance stress, can be generated randomly and saved in a memory location. It is mandatory to retrieve the selected *SEED${}_{IN}$* since it must be used to reconstruct the data sequence during a read operation;
2. Download the content of ALFSR${}_{1}$ into ALFSR${}_{2}$. In practice, the Seed of ALFSR${}_{2}$ is the present state of ALFSR${}_{1}$;
3. Program the memory page by cycling ALFSR${}_{2}$ until the completion of the page programming while keeping ALFSR${}_{1}$ on hold, preventing an evolution of its internal state;
4. Send a clock pulse to ALFSR${}_{1}$ that moves to the next state;
5. Repeat steps 2 to 4 until the completion of the block programming.

**Figure 8:**
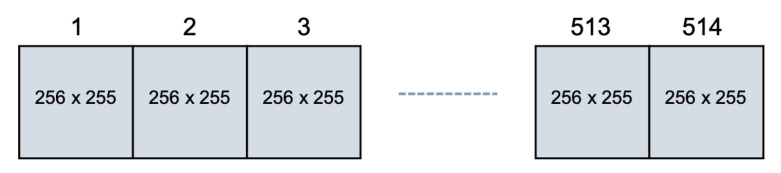
When applied to a $N_{P}$ = 256 ×$N_{B}$ = $2^{17}$ memory block, the data provided by an 8-bit ALFSR consist of a sequence of 256 × 255 arrays.

By considering a $N_{P}$ = 256 ×$N_{B}$ = $2^{17}$ memory block and an 8-bit ALFSR, the data provided by the proposed method consist of a sequence of 514 pseudo symmetrical arrays, each with 256 rows and 255 columns, as shown in Figure 8. In any array, the statistical properties provided by the 8-bit ALFRS are guaranteed. Since the ALFSR period is $2^{k}-1$, we can avoid issues related to the logical period that are powers of two.

A *k*-bit counter is required if the memory block is not programmed sequentially page after page. The counter is preset with the index of the page to be programmed. ALFSR${}_{1}$ is initialized with *SEED${}_{IN}$* as in point 1 of the described algorithm. Then a countdown starts and, with every clock pulse, ALFSR${}_{1}$ moves to the next state. When the counter reaches the zero state, the content of ALFSR${}_{1}$ is downloaded to ALFSR${}_{2}$ (as in point 2 of the algorithm) since it represents the correct Seed for the page to be programmed.

When the memory block is read sequentially page after page, the same procedure used for data programming is applied. When a single page is to be read, the procedure used for non-sequential programming is applied with the counter initialized with the page address.

**Figure 9:**
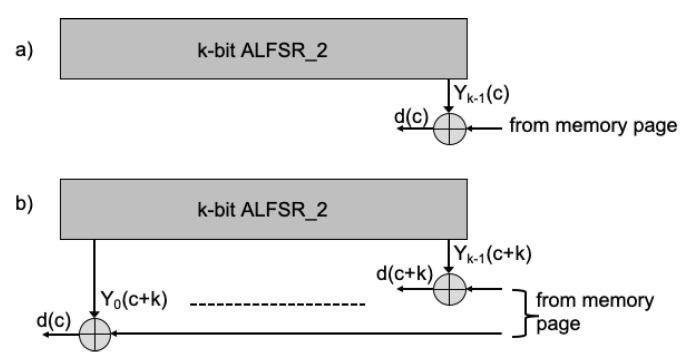
Possible schemes used to reconstruct data read from a memory page. (**a**) At any clock cycle *c* the read data is XORed with the last register bit $Y_{k-1}\left(c\right)$ to provide d(c); (**b**) *k* clock pulses are applied to ALFSR${}_{2}$, then the register content is XORed with *k* data read and the procedure is repeated until all page data are read.

Data stored in the memory array can be easily reconstructed by XORing the data saved in the memory cells with the ALFRS${}_{2}$ content, depending on the randomizing scheme (see Figure 9).

## 4. Experimental Validation

The experimental validation of a randomization scheme requires the assessment of the memory reliability according to the data pattern written within. In this work, this activity took place by characterizing the RBER of an off-the-shelf *N* (N<100) layers TLC 3D NAND Flash technology featuring *M* (M<16) wordlines per layer, where its input data were supplied either by a Horizontal Centric (HC) randomizer (i.e., a randomizer that does not properly control the vertical randomization) or by our proposed method. The RBER characterization has been performed in two well-defined corners of the memory lifetime, namely after an endurance stress test (i.e., repeatedly writing and erasing the memory blocks) and after a data retention test at high temperatures. The standards adopted for endurance stress and data retention were chosen according to the JEDEC tests specifications for the 3D NAND Flash enterprise qualification procedure [19], resulting in 3000 Program/Erase cycles (that is the technology rated endurance) at a temperature of 61 ${}^{\circ }$C for 500 h cycle time and a retention stress test performed on cycled devices for 3 months at 40 ${}^{\circ }$C.

The experimental setup described in [20] has been exploited for both characterizations. To rule out any topological artifacts in the measurements, we disabled any error correction functionality of the chip, and we did not apply any modification to the standard working voltages of the devices and no special test modes were exploited to filter the RBER. We also ruled out the presence of on-chip randomizer circuitry that could alter the findings. The data analysis was performed on all the wordlines within a memory block considering all the TLC page types. The size of a page is 16 kB, along with the parity left for error correction code purposes, divided into 4 kB chunks, which are the minimum units read during tests by the characterization system. The testing lasted several months.

**Figure 10:**
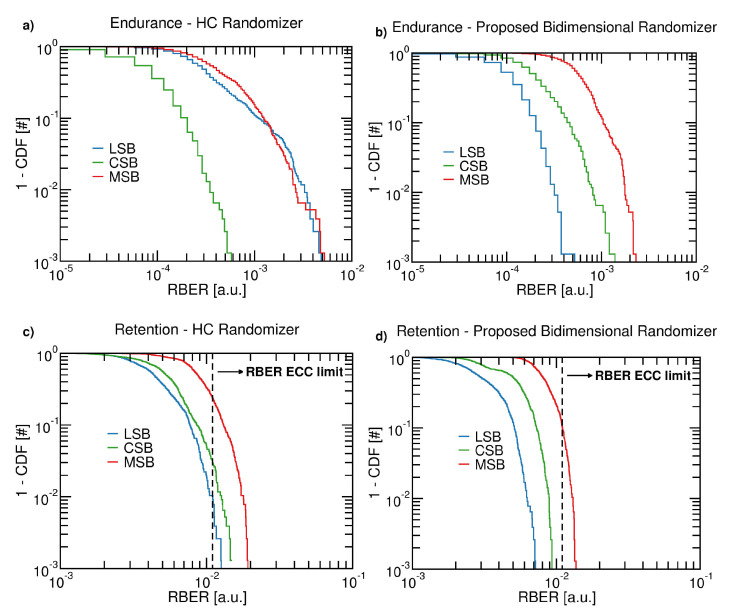
Complementary Cumulative Distribution Function (1-CDF) of the RBER in a 3D NAND Flash memory for Endurance and Retention working corners when the input data come from an HC randomizer (**a**–**c**) or from our proposed method (**b**–**d**).

Figure 10 shows the Complementary Cumulative Distribution Function (1-CDF) as a function of the TLC page type of the RBER in a 3D NAND Flash block programmed with an HC randomizer (cases *a* and *c*) or with our proposed method (cases *b* and *d*). For the endurance stress cases we observe that an HC randomizer could dangerously induce an RBER close to the error correction capacity of many advanced schemes (we refer here to the case of a 100 b/1 kB that is a maximum allowable RBER of 1.1 × 10${}^{-2}$) [21,22], whereas with our proposed method, we still have a sufficient margin with respect to that reliability limit. In the HC randomizer, we remember that there is neither a control of the patterns of ones and zeros achieved along the bitline (i.e., vertical dimension) nor an upper bound to their maximum length. This can result in some fortunate patterns (as observed in these experiments for the CSB pages), where the RBER appears as the best; however, this is at the expense of inducing the worst patterns on the other TLC pages (i.e., LSB and MSB). From the statistical standpoint, we observe an imbalanced situation where the optimal patterns are concentrated only in one TLC page type. With our proposed methodology, we guarantee a good pattern balancing among the TLC pages, thus significantly reducing the worst RBER case and homogenizing the behavior of all the 3D NAND Flash memory locations. This leads to a better control of the 3D NAND Flash reliability and in turn to a reduced system-level effort in coping with endurance and retention errors using complex error correction codes or secondary correction schemes. We also want to stress that, due to the different architectural and integration options of 3D NAND Flash technology [17], we expect that the RBER behavior may expose a different trend for memories manufactured with a different process/architecture with respect to that characterized in this work. However, our proposed randomization methodology is still foreseen to yield the same RBER improvements, while the HC randomizer will still evidence shortcomings in terms of the worst case RBER.

If we consider the retention test case, which is an additive RBER factor with respect to what we observed during the endurance test (retention tests are performed after that), we observe that all TLC pages written with an HC randomizer become uncorrectable since their RBER crosses the 1.1 × 10${}^{-2}$ limit, whereas in our method, only the MSB pages are above it. This suggests that, while with our method we may think to develop secondary error correction schemes [23,24,25] targeted only at MSB pages in retention conditions, with an HC randomizer we are forced to deal with an additional effort to recover the data every time we access the 3D NAND Flash after a data retention stress.

**Figure 11:**
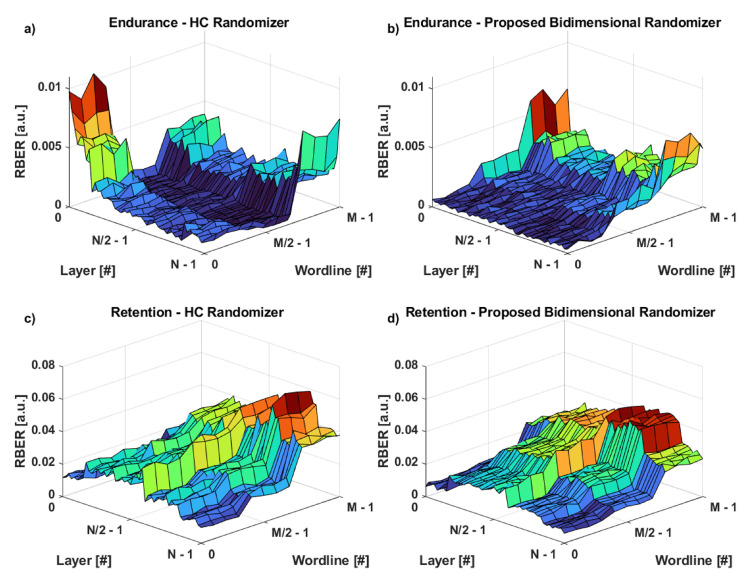
Topological characterization of the RBER in a 3D NAND Flash memory for Endurance and Retention working corners when the input data come from an HC randomizer (**a**,**c**) or from our proposed method (**b**,**d**).

Finally, Figure 11 shows the results of a topological characterization of the RBER in a 3D NAND Flash block. As can be seen, there are specific areas (i.e., layers and wordlines) for which the use of a good quality randomizer could help in terms of improving the reliability during both endurance and retention working conditions. We must note that, in the HC randomizer, the presence of uncontrolled sequences of consecutive ones and zeros, coupled with the non-perfect randomization along the vertical dimension of the memory, severely affects the sensing operation of the data with the consequent burden on the RBER. In 3D NAND Flash memory architectures (please refer to Figure 1a), the layers are the contacts stacked along the vertical dimension (let us refer to it as the y-axis) also referred to as the control gates to which the voltages for programming and reading the memory are applied. For each layer, there are several wordlines associated and connected in the direction of the z-axis, so that a single layer (control gate) can drive the signal in parallel on multiple wordlines. The bitlines are in the x-axis direction. Let us assume a total of five wordlines per layer. Since we have a TLC storage paradigm, we will come up with five wordlines associated with LSB, five wordlines for CSB, and five wordlines for MSB. That is why we have well-defined “stripes” in the plots of Figure 11. The higher wordline indexes are associated with MSB pages and the lower indexes to LSB. In this case, Figure 11 reflects the same behavior as observed in Figure 10. Concerning the variability of RBER characteristics along the layers and the wordlines, we must note that the 3D NAND Flash manufacturing process is not easy to control, so there is a well-known sensitivity of the RBER’s layers that depends exactly on the peculiar processing steps of the memory devices, which, unfortunately, are not disclosed to us.

## 5. Conclusions

In this work, we proposed a randomization scheme for 3D NAND Flash memory technology that allows a good degree of randomization in both memory dimensions (i.e., wordline and bitline) without requiring a complex implementation methodology while relying only on a proper arrangement of LFSRs circuits. The simulation results show that our methodology imposes a guard band on the maximum number of consecutive ones and zeros along the bitline dimensions (no more than 25) to keep the read failure probability during the data readout phase under control.

Further, we demonstrate by construction that our randomization scheme has better control of the number of zeros or ones along the bitline, proving a good balancing of the write data to the memory, thus representing an optimal case for reliability.

Finally, we experimentally validated our proposed methodology on an off-the-shelf TLC 3D NAND Flash memory chip, showing that, under JEDEC-style endurance and data retention stress, we can achieve RBER for LSB and CSB pages that is always below the correction limit imposed by a 100 b/1 kB Error Correction Code. Our randomization methodology can therefore be exploited by storage system designers to keep the memory reliability under control.

## Figures and Tables

**Table 1:** Each row shows the sequence of $2^{4}-1$ bits $Y_{3}\left(c\right)$ generated by a 4-bit ALFSR whose initial seed is selected randomly. The 4th column enlightens the presence of long sequences of 0 or 1.

0	0	0	**1**	0	0	1	1	0	1	0	1	1	1	1
1	0	0	**1**	1	0	1	0	1	1	1	1	0	0	0
0	0	1	**1**	0	1	0	1	1	1	1	0	0	0	1
1	1	0	**1**	0	1	1	1	1	0	0	0	1	0	0
0	1	0	**1**	1	1	1	0	0	0	1	0	0	1	1
1	0	1	**1**	1	1	0	0	0	1	0	0	1	1	0
0	1	1	**1**	1	0	0	0	1	0	0	1	1	0	1
1	1	1	**1**	0	0	0	1	0	0	1	1	0	1	0
1	0	0	**0**	1	0	0	1	1	0	1	0	1	1	1
0	0	1	**0**	0	1	1	0	1	0	1	1	1	1	0
0	1	0	**0**	1	1	0	1	0	1	1	1	1	0	0
0	1	1	**0**	1	0	1	1	1	1	0	0	0	1	0
1	0	1	**0**	1	1	1	1	0	0	0	1	0	0	1
1	1	1	**0**	0	0	1	0	0	1	1	0	1	0	1
1	1	0	**0**	0	1	0	0	1	1	0	1	0	1	1

**Table 2:** The first row shows the sequence of $2^{4}-1$ bits $Y_{3}\left(c\right)$ generated by a 4-bit ALFSR whose initial seed is selected randomly. Each following row is equal to the previous one left-shifted by 1 position. The resulting array is symmetrical since, by construction, any *i*th row and column are identical (for instance, the 5th row and column are enlightened).

0	0	1	0	0	1	1	0	1	0	1	1	1	1	0
0	1	0	0	**1**	1	0	1	0	1	1	1	1	0	0
1	0	0	1	**1**	0	1	0	1	1	1	1	0	0	0
0	0	1	1	**0**	1	0	1	1	1	1	0	0	0	1
**0**	**1**	**1**	**0**	**1**	**0**	**1**	**1**	**1**	**1**	**0**	**0**	**0**	**1**	**0**
1	1	0	1	**0**	1	1	1	1	0	0	0	1	0	0
1	0	1	0	**1**	1	1	1	0	0	0	1	0	0	1
0	1	0	1	**1**	1	1	0	0	0	1	0	0	1	1
1	0	1	1	**1**	1	0	0	0	1	0	0	1	1	0
0	1	1	1	**1**	0	0	0	1	0	0	1	1	0	1
1	1	1	1	**0**	0	0	1	0	0	1	1	0	1	0
1	1	1	0	**0**	0	1	0	0	1	1	0	1	0	1
1	1	0	0	**0**	1	0	0	1	1	0	1	0	1	1
1	0	0	0	**1**	0	0	1	1	0	1	0	1	1	1
0	0	0	1	**0**	0	1	1	0	1	0	1	1	1	0

## Abbreviations

The following abbreviations are used in this manuscript:

MMC	MultiMedia Card
SSD	Solid State Drive
RBER	Raw Bit Error Rate
LFSR	Linear Feedback Shift Register
LUT	Look Up Table
